# Functional Assessment of Coding and Regulatory Variants From the *DKK1* Locus

**DOI:** 10.1002/jbm4.10423

**Published:** 2020-11-02

**Authors:** Núria Martínez‐Gil, Neus Roca‐Ayats, Nurgül Atalay, Marta Pineda‐Moncusí, Natàlia Garcia‐Giralt, Wim Van Hul, Eveline Boudin, Diana Ovejero, Leonardo Mellibovsky, Xavier Nogués, Adolfo Díez‐Pérez, Daniel Grinberg, Susanna Balcells

**Affiliations:** ^1^ Department of Genetics, Microbiology and Statistics, Faculty of Biology Universitat de Barcelona, Centro de Investigación Biomédica en Red en Enfermedades Raras (CIBERER), Institut de Biomedicina de la Universitat de Barcelona (IBUB), Institut de Recerca Sant Joan de Déu (IRSJD) Barcelona Spain; ^2^ Musculoskeletal Research Group, Hospital del Mar Medical Research Institute Centro de Investigación Biomédica en Red en Fragilidad y Envejecimiento Saludable, ISCIII Barcelona Spain; ^3^ Center of Medical Genetics University of Antwerp & University Hospital Antwerp Antwerp Belgium

**Keywords:** WNT/β‐CATENIN/LRPs, CELLS OF BONE OSTEOBLASTS, BONE DISEASES AND DISORDERS, OSTEOPOROSIS, GENETIC RESEARCH, MOLECULAR PATHWAYS REMODELING

## Abstract

The *DKK1* gene encodes an extracellular inhibitor of the Wnt pathway with an important role in bone tissue development, bone homeostasis, and different critical aspects of bone biology. Several BMD genome‐wide association studies (GWASs) have consistently found association with SNPs in the *DKK1* genomic region. For these reasons, it is important to assess the functionality of coding and regulatory variants in the gene. Here, we have studied the functionality of putative regulatory variants, previously found associated with BMD in different studies by others and ourselves, and also six missense variants present in the general population. Using a Wnt‐pathway‐specific luciferase reporter assay, we have determined that the variants p.Ala41Thr, p.Tyr74Phe, p.Arg120Leu, and p.Ser157Ile display a reduced DKK1 inhibitory capacity as compared with WT. This result agrees with the high‐bone‐mass (HBM) phenotype of two women from our cohort who carried mutations p.Tyr74Phe or p.Arg120Leu. On the other hand, by means of a circularized chromosome conformation capture‐ (4C‐) sequencing experiment, we have detected that the region containing 24 BMD‐GWA variants, located 350‐kb downstream of *DKK1*, interacts both with *DKK1* and the *LNCAROD* (LncRNA‐activating regulator of DKK1, AKA *LINC0148*) in osteoblastic cells. In conclusion, we have shown that some rare coding variants are partial loss‐of‐function mutations that may lead to a HBM phenotype, whereas the common SNPs associated with BMD in GWASs belong to a putative long‐range regulatory region, through a yet unknown mechanism involving *LNCAROD*. © 2020 The Authors. *JBMR Plus* published by Wiley Periodicals LLC on behalf of American Society for Bone and Mineral Research.

## Introduction

For the last two decades, genome‐wide association studies (GWASs) have been a powerful tool to identify genes associated with complex diseases. Regarding osteoporosis, more than 500 loci associated with BMD have been determined. However, these loci only explain 20% of the BMD variability; few have been attributed a clear functional mechanism.^(^ ^1^ ^)^ The challenge of current genetics is to establish the underlying functional basis of all these associations.

One of the most enriched pathways in genome‐wide significant signals for BMD is the Wnt pathway. Polymorphisms in several of its genes have been associated with BMD and fracture risk.^(^ ^1^, ^2^, ^3^, ^4^, ^5^, ^6^, ^7^, ^8^, ^9^, ^10^ ^)^ Moreover, rare and penetrant mutations causing Mendelian bone phenotypes have been described in Wnt pathway genes, such as the loss‐of‐function and gain‐of‐function mutations in the LRP5 coreceptor, causing the two diametrically opposed phenotypes of osteoporosis‐pseudoglioma syndrome^(^ ^11^ ^)^ and high bone mass (HBM),^(^ ^12^, ^13^, ^14^ ^)^ respectively. The Wnt pathway is finely regulated by several extracellular inhibitors, among which sclerostin and DKK1 stand out. These two proteins prevent the formation of the heterotrimeric complex LRP5/6‐FZD‐Wnt^(^ ^15^ ^)^ by forming other heterotrimeric complexes, together with LRP5 and LRP4 (in the case of sclerostin)^(^ ^16^, ^17^ ^)^ or with LRP5 and Kremen1/2 (in the case of DKK1).^(^ ^18^ ^)^ DKK1 binds both the first and third β‐propeller domains of LRP5,^(^ ^19^, ^20^ ^)^ and it is in the first β‐propeller domain where mutations causing the HBM phenotype are clustered.^(^ ^21^, ^22^, ^23^ ^)^ Different studies have highlighted the important role of the DKK1 protein in the development of skeletal tissue, in bone homeostasis and in different critical aspects of bone biology.^(^ ^15^, ^24^, ^25^ ^)^ In mice, the homozygous *Dkk1* KO is lethal at birth and displays severe defects in head formation that result in anterior truncations.^(^ ^26^ ^)^ In contrast, the heterozygous KO, the hypomorphic (doubleridge), the tamoxifen inducible at 7 weeks, or the osteolineage‐specific mouse models show an increase in BMD and an increase in bone formation.^(^ ^27^, ^28^, ^29^, ^30^, ^31^ ^)^ By studying the allelic combination of null or doubleridge mice (Dkk1+/db; Dkk1+/−; Dkk1db/db; Dkk1db/−; Dkk1−/−), a gene dose‐dependent inverse correlation with BMD was observed.^(^ ^28^, ^29^ ^)^ On the other hand, transgenic overexpression of the *Dkk1* gene in osteoblasts produces a relative decrease in the number of osteoblasts, thus producing a decrease in bone formation.^(^ ^32^, ^33^ ^)^ In the last decade, thanks to the direct effect on the inhibition of osteoblastogenesis and the indirect activation of osteoclastogenesis, sclerostin and DKK1 have become interesting targets for the anabolic treatment of osteoporosis. In particular, monoclonal antibodies against DKK1 stimulate bone formation in younger animals and to a lesser extent in adult animals, and enhance fracture healing.^(^ ^34^ ^)^

Given the importance of DKK1 in bone physiology and pathology, it is essential to reach a comprehensive understanding of the functional roles of the *DKK1* variants. In a previous study, we resequenced the transcribed regions and the intronic flanks of the gene in two patient groups with opposed and extreme BMD values from the Barcelona osteoporosis (BARCOS) cohort of postmenopausal women, and found three interesting variants in potentially functional regions: a rare missense variant (rs149268042; p.Arg120Leu), a rare 3′UTR (untranslated region) variant (rs74711339), and a common variant predicted to affect splicing (rs1569198), which was also found nominally associated with femoral neck BMD in the complete cohort.^(^ ^35^ ^)^ In addition to these variants, a region between *DKK1* and *MBL2* is relevant because many GWASs on BMD found strong associations with SNPs clustered in it and not with SNPs inside the *DKK1* gene.^(^ ^1^, ^3^, ^4^, ^5^, ^36^, ^37^, ^38^, ^39^, ^40^, ^41^ ^)^

**Fig 1:**
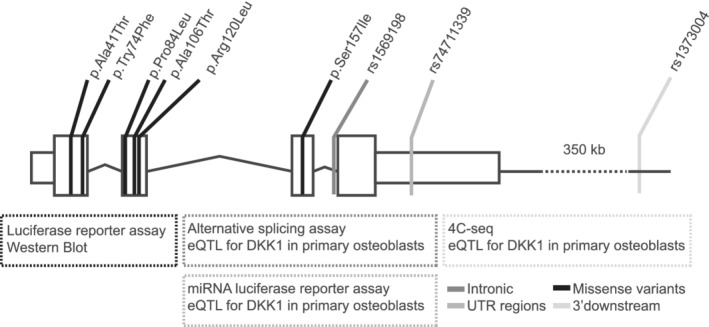
Scheme of the *DKK1* gene including coding exons (boxes), introns (lines), 5′ and 3′UTR (small boxes) and the location of all the variants tested. Experiments performed to test the functionality of each type of variant are indicated below the scheme. eQTL = Expression quantitative trait loci; miRNA = microRNA; UTR = untranslated region.

In this study, our objective was to test the underlying functionality of selected variants found in *DKK1*, as well as to determine if the region harboring the genome‐wide significant SNPs is actually regulating the *DKK1* gene, the *MBL2* gene, or both. We carried out functional studies specific for each variant (Fig. 1), and a 4C‐seq experiment to study a GWA‐significant SNP‐rich region. With this, we determined that the missense variants p.Ala41Thr, p.Tyr74Phe, p.Arg120Leu, and p.Ser157Ile reduce the DKK1 inhibitory capacity, and that the GWA‐SNP region is indeed interacting with *DKK1* and also with the neighboring lncRNA *LNCAROD* (AKA *LINC0148*) gene.

## Materials and Methods

### Cell culture

The human osteosarcoma cell line Saos‐2 was used for luciferase reporter assays and 4C‐seq assays. It was obtained from the American Type Culture Collection (ATCC HTB‐85) and grown in DMEM (Sigma‐Aldrich, St. Louis, MO, USA), supplemented with 10% FBS (Gibco–Life Technologies, Grand Island, NY, USA) and 1% penicillin/streptomycin (p/s; Gibco–Life Technologies), at 37°C and 5% of CO_2_. The human fetal osteoblast 1.19 (hFOB) cell line and human medulla‐derived mesenchymal stem cells (MSCs) were used for 4C‐seq assays. The hFOB 1.19 cell line was obtained from ATCC (ATCC CRL‐11372) and grown in DMEM:F12 (1:1) medium without phenol red (Gibco–Life Technologies), supplemented with 10% FBS and 0.3 mg/mL geneticin (Gibco–Life Technologies) at 34°C and 5% of CO_2_. MSCs were kindly provided by Dr. José Manuel Quesada Gómez from Instituto Maimónides de Investigación Biomédica, Hospital Universitario Reina Sofía, Córdoba, Spain.^(^ ^42^ ^)^ These cells were grown in α‐MEM medium (Gibco–Life Technologies), supplemented with 10% FBS, 1% p/s, and 1x glutamax (Gibco–Life Technologies) at 37°C and 5% of CO_2_. Human primary osteoblasts (hOBs) used for expression quantitative trait loci (eQTLs) assays are described in Roca‐Ayats and colleagues.^(^ ^43^ ^)^

### Luciferase reporter constructs and *DKK1* site‐directed mutagenesis

The mouse *Wnt1*‐V5 expression vector, *mesdc2* expression vector, human WT *LRP5* expression vector, pRL‐TK, pGL3‐OT reporter, and human *DKK1*‐FLAG expression vector are described in Balemans and colleagues.^(^ ^44^ ^)^ The mutations p.Ala41Tyr, p.Tyr74Phe, p.Pro84Leu, p.Ala106Thr, p.Arg120Leu, and p.Ser157Ile were introduced into the expression vector *DKK1*‐FLAG using the QuickChange Site‐Directed Mutagenesis kit (Agilent Technologies, Santa Clara, CA, USA), following the manufacturer's instructions. A 300‐bp fragment of the DKK1 3′UTR containing the SNP rs74711339 in a central position was cloned (in each of the two allele versions) into the pmirGLO dual‐luciferase miRNA target expression vector (Promega, San Luis Obispo, CA, USA). Constructs were cloned using the XhoI and SdaI restriction sites. All primers used are detailed in Supplementary Information Table S1. In all cases, the presence of point mutations and absence of errors were verified through Sanger sequencing.

### Luciferase gene reporter assay

Two luciferase gene reporter assays were performed: one to test the inhibitory activity of WT and mutant DKK1 proteins (Wnt pathway reporter assay) and the other to test the effect of the 3′UTR variant rs74711339 (3′UTR reporter assay). For both experiments, 3 × 10^5^ Saos‐2 cells per well were cultured in 6‐well plates, 24 hours before the transfection. For the Wnt pathway reporter assay, we cotransfected: mouse *Wnt1*‐V5 (64 ng), *mesdc2* (128 ng), human WT *LRP5* (128 ng), pRL‐TK (160 ng), pGL3‐OT reporter (1600 ng). and DKK1 (48 ng). When necessary, the empty pcDNA3 vector was used to equalize the total amount of DNA transfected in each experiment (total DNA = 2128 ng). For the miRNA reporter assay, we transfected at a 1:1 ratio, the pGFP vector and either the empty pmirGLO or with the DKK1‐3′UTR fragment with one or the other allele (total DNA =1.6 μg). Fugene HD was used following the manufacturer's instructions. Forty‐eight hours after transfection, cells were lysed and the luciferase activities of *Photinus pyrali* and *Renilla reniformis* were measured using a Glomax Multi + luminometer (Promega), following the instructions of the dual‐luciferase reporter assay system (Promega).

### Western blot assay

For the Western blot assay, 3 × 10^5^ Saos‐2 cells per well were cultured in 6‐well plates, 24 hours before the transfection. We transfected 2 μg of the DKK1 expression plasmid (WT or the missense variant: p.Ala41Thr, p.Tyr74Phe, p.Pro84Leu, p.Ala106Thr, p.Arg120Leu, p.Ser157Ile). For the negative control, we transfected 2 μg of the empty pcDNA3 vector. Fugene HD was used following the manufacturer's instructions. After 24 hours, the medium was changed, reducing from 2 to 1 mL of DMEM, without FBS or antibiotics. Forty‐eight hours after transfection, the supernatant (conditioned medium) of each condition was collected and were concentrated using 10 K Amicon Ultra filters (Millipore, Watford, UK). Extracellular proteins (concentrated conditioned medium) were quantified using the Pierce BCA Protein Assay kit (Thermo Fisher Scientific, Waltham, MA, USA). Proteins from concentrated conditioned medium (5 μg/lane) were separated by electrophoresis in an SDS‐PAGE and transferred to a hydrophobic polyvinylidene fluoride transfer membrane (Millipore). The ab109416 antibody against DKK1 (Abcam, Cambridge, UK) was used. The images were developed using a peroxidase‐conjugated secondary antibody (anti‐rabbit IgG; A0545) for DKK1 antibody. All the experiments were performed in three independent biological replicates.

### eQTL assay

DNA was extracted from cultured hOBs using the Wizard Genomic DNA Purification kit (Promega), according to the manufacturer's instructions. The concentration of the purified DNA was analyzed on a spectrophotometer (NanoDrop; Thermo Fisher Scientific). Genotypes for rs1569198, rs74711339, and rs1373004 were assessed by Sanger sequencing using the BigDye Terminator v3.1 (Applied Biosystems, Warrington, UK) in the genomics facilities of the Universitat de Barcelona. Primers (Invitrogen, Carlsbad, CA, USA; Thermo Fisher Scientific) were designed using the Primer3 Input 0.4.0 (Supplementary Information Table S1). RNA was extracted from cultured hOBs using the High Pure RNA Isolation kit (Roche Diagnostics, Mannheim, Germany), according to the manufacturer's instructions. RNA was quantified using a NanoDrop spectrophotometer and retrotranscribed using the High‐Capacity cDNA Reverse Transcription kit (Applied Biosystems, Thermo Fisher Scientific), according to the specifications of the manufacturer. qPCR was performed using UPL probes (Roche) on a LightCycler 480 Instrument II (Roche). *HMBS* gene expression was used as normalizing control, and fold changes were calculated by relative quantification, using the second derivative method. Primers are summarized in Supplementary Information Table S1.

### Alternative splicing analysis

hOB cDNA from homozygous women for the rs1569198 SNP was amplified using two primers (Fw: TCCGAGGAGAAATTGAGGAA and R: TCCATGAGAGCCTTTTCTCC). The forward primer is located at the 3′end of exon 3 and the reverse primer is located at the 5′ end of exon 4. This pair of primers would amplify a 254‐bp amplicon in the case of WT splicing, and a 296‐bp amplicon in the case of alternative splicing.

### Circularized chromosome conformation capture sequencing

Circularized chromosome conformation capture‐sequencing (4C‐seq) experiment was carried out at the Functional Genomics Service of the Centro Andaluz de Biología del Desarrollo (Sevilla, Spain). 4C‐seq libraries were generated from Saos‐2, hFOB 1.19 and hMSC cell lines as described previously.^(^ ^45^, ^46^ ^)^ 4‐bp cutters were used as primary (DpnII) and secondary (Csp6I) restriction enzymes. For each cell line, a total of 1.6 μg of library was PCR amplified (primers used: AGTAAGCTGTGGAATCAATGAC and CTGAGCCTCTCTTCTCGGATC, chr10:54427977–54428133, GRCh37). Samples were sequenced with Illumina Hi‐Seq technology according to standard protocols at the Genomics Service of the Centro Nacional de Investigaciones Cardiovasculares (CNIC, Madrid, Spain). 4C‐seq data were generated as described previously.^(^ ^47^ ^)^ Briefly, raw sequencing data were demultiplexed and mapped to the corresponding reference genome (GRCh37). Reads located in fragments flanked by two restriction sites of the same enzyme, in fragments smaller than 40 bp or within a window of 10 kb around the viewpoint were filtered out. 4C‐seq data were normalized by the total weight of reads within ±2 Mb around the viewpoint.

4C‐seq data were analyzed with the AFourC software (publicly available at https://github.com/Nikoula86/AFourC.git), following and adapting previously described pipelines.^(^ ^48^, ^49^, ^50^ ^)^ Briefly, we assumed that the 4C signal profile relative to the viewpoint *v* with coordinate *xv* on chromosome *N* is formed by three independent contributions: a constant background level, a negative exponential representing the monotonic decay of the 4C signal with the genomic distance from the viewpoint,^(50^ ^)^ and *N* gaussians representing the interaction peaks:

S4Cx=B+Ixv∙e−x−xvλ+∑i=1NPxi∙e−x−xi22∙σ2

To estimate the genomic distance‐dependent monotonic decay, we assumed a symmetric trend around the viewpoint and performed the exponential fit on the left–right averaged profile. Statistically significant peaks were detected using a *p* value of 0.0005.

### Statistical methods

#### Wnt pathway assay

The ratio of the Firefly and Renilla luciferase measurement was calculated (relative luciferase units; RLUs). For each mutant, the test factor had the following levels: Empty (refers to the luciferase activity resulting from the endogenous Wnt pathway), Active (luciferase activity produced by the Wnt pathway in the presence of exogenous Wnt1, medc2, and LRP5), Inhibitor (luciferase activity in the presence of exogenous Wnt1, mesdc2, LRP5, and WT DKK1 inhibitor), and Mutant (luciferase activity in the presence of exogenous Wnt1, mesdc2, LRP5, and each of the mutant DKK1 proteins). We analyzed the data distribution and heteroscedasticity via the Shapiro‐Wilk test for normality and the Breusch‐Pagan test, respectively. We normalized replicates per day using the DKK1‐WT inhibition to reduce the nuisance factor. Then, we assessed the differences between luciferase activities through the Kruskal‐Wallis test. A post hoc test for multiple comparisons of groups was performed according to pairwise comparisons using the Wilcoxon rank sum test, adjusting *p* values with Bonferroni. Statistical analyses were performed using R software v. 3.5.3 (R Foundation for Statistical Computing, Vienna, Austria; https://www.r-project.org/), and *p* < 0.05 was considered significant. GraphPad (GraphPad Prism 8; GraphPad Software, La Jolla, CA, USA) was used for graphic representation. Each experiment included a minimum of three replicates and was repeated independently in three separate experiments.

#### 3′UTR assay

The ratio of the Firefly and Renilla luciferase measurement was calculated (RLUs). We analyzed the data distribution and homogeneity of variances via the Shapiro‐Wilk test for normality and the Bartlett test, respectively. We normalized replicates per day using the empty vector activity to reduce the nuisance factor. We assessed the differences between luciferase activities through an ANOVA test. The Tukey HSD (honestly significant difference) test was used to perform the post hoc test for multiple group comparisons, testing the effect between all the conditions in the miRNA assay. Statistical analyses were performed using R software v. 3.5.3 (R Foundation for Statistical Computing), and *p* < 0.05 was considered significant. GraphPad (Graphpad Prism 8) was used for graphic representation. Each experiment included three replicates and was repeated independently in four separate experiments.

#### eQTL assay

For the study of the eQTLs, the WGassociation function in R software v.3.5.3 was used.^(^ ^51^ ^)^ This function carries out an association analysis between SNPs and a dependent variable (DKK1 expression levels) under five different genetic inheritance models: codominant, dominant, recessive, overdominant, and log‐additive.

## Results

### Functional evaluation of six *DKK1* missense variants

**Fig 2:**
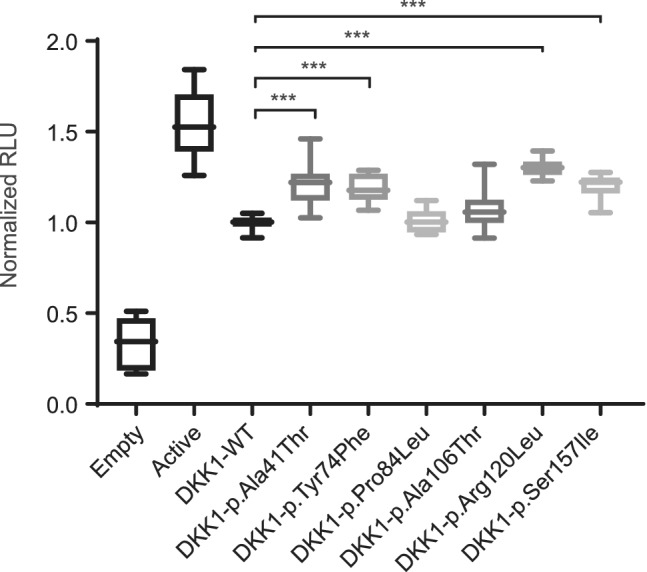
Luciferase reporter assays of six DKK1 missense variants. Boxplots of the normalized relative luciferase activity (RLU) of the following transfections: endogenous Wnt pathway (transfected only with the TCF/LEF reporter construct and the empty pcDNA3 vector; *Empty*), activated pathway (cotransfection: same as before plus Wnt1, LRP5 and mesdc2; *Active*) and the pathway inhibited by either the WT DKK1 or the six mutated DKK1 proteins (cotransfection as before plus the corresponding DKK1 constructs). Significant differences between the mutated DKK1 and the WT DKK1 (used as normalizer) are indicated as ***p < 0.001. Error bars represent the SD.

To measure the inhibitory activity of six mutant DKK1 proteins (Fig. 1) on the canonical Wnt pathway, we performed a specific luciferase reporter gene assay. The transfection of *Wnt1, mesdc2*, and *LRP5*, together with a TCF/LEF‐luciferase reporter, in Saos‐2 cells resulted in an approximate fivefold stimulation of the endogenous Wnt pathway as previously described.^(^ ^22^ ^)^ Likewise, addition of WT DKK1 resulted in a one‐third inhibition of the pathway (35.2%; Fig. 2). In contrast, addition of either of the four DKK1 mutant proteins, p.Ala41Thr, p.Tyr74Phe, p.Arg120Leu, and p.Ser157Ile, resulted in a significantly reduced inhibitory capacity as follows: p.Ala41Thr displayed a 21.9% inhibition of the fully stimulated Wnt pathway (37.8% loss of the inhibition related to WT DKK1; *p* < 0.001), p.Tyr74Phe, a 23.2% inhibition of the Wnt pathway (34% of WT DKK1; *p* < 0.001), p.Arg120Leu, a 15.6% inhibition (55.2% of WT DKK1; *p* < 0.001), and p.Ser157Ile, a 22.2% inhibition (37% of WT DKK1; *p* < 0.001). No significant differences were observed for the remaining two missense variants (Fig. 2). By performing Western blot analyses of the different DKK1 mutant proteins secreted to the extracellular space, we observed that only the p.Ser157Ile mutant was expressed at lower levels than those of WT DKK1. In contrast, all other mutants were expressed at similar or higher levels compared to the WT (Supplementary Information Fig. S1). Altogether, we have determined that four of the six missense variants do affect the DKK1 inhibitory capacity.

### Functional evaluation of variants rs74711339 (3′UTR) and rs1569198 (intronic)

To test the functional activity of the *DKK1*‐3′UTR variant rs74711339, we performed a 3'UTR‐luciferase reporter assay in Saos‐2 cells. Significant differences were found between the empty vector and either allele, whereas no significant differences were detected between both alleles (Supplementary Information Fig. S2). This result indicates that 3′UTR of *DKK1* is involved in transcript expression or stabilization in Saos‐2 cells, but the SNP tested is not affecting this regulation.

Variant rs1569198 is located in intron 3, 43 nucleotides upstream of the last exon of the gene. According to several in silico predictors, the G allele would generate a new splicing acceptor site (atgcaggttta**g**CA), producing a protein with 16 additional amino acids, encoded by the 3′ end of intron 3. However, this alternative transcript has not been detected in primary osteoblast cDNA from 12 women homozygous for the G allele (Supplementary Information Fig. S3).

SNPs rs1569198 and rs74711339 are defined as eQTLs of *DKK1* in adrenal gland and transformed fibroblasts, respectively, according to GTEx (V8 release; Supplementary Information Table S2). Because bone tissue is not available in this database, we used an in‐house collection of 45 primary osteoblasts to determine whether these two SNPs (plus rs1373004, found associated with BMD and risk of fracture in Estrada and colleagues^(^ ^3^ ^)^) are *cis*‐eQTLs of *DKK1* in these cells. None of the tested SNPs resulted as eQTLs in our samples (Supplementary Information Fig. S4).

### Chromatin interaction from the GWAS hit locus

**Fig 3:**
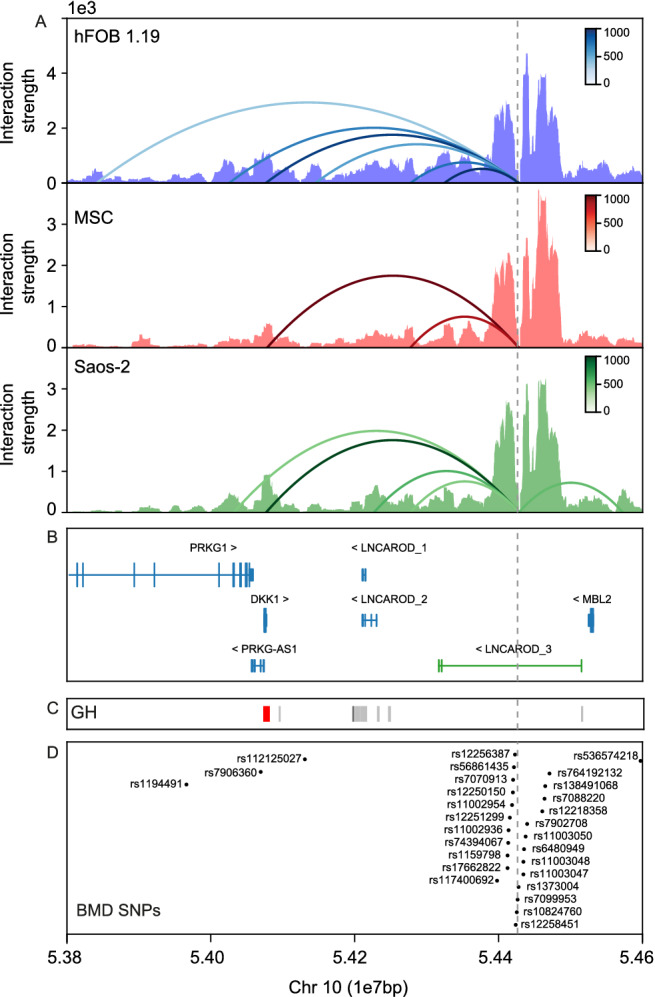
4C‐seq using the SNP rs1373004 region as viewpoint in human fetal osteoblast (hFOB 1.19), mesenchymal stem cells (MSCs), and Saos‐2 cell line. (*A*) Graphical representations of the read depths (Interaction strength) across the *DKK1‐MBL2* genomic region. Significant interactions are marked with arched lines and the color scale corresponds to the strength of these significant interactions. (*B*) Genes in this region: *PRKG1* (ENST00000373980.4), *PRKG1‐AS1* (NR_038277.1), *DKK1* (ENST00000373970.3), *LNCAROD_1* (NR_120642.1), *LNCARDOD_2* (NR_120641.1), and *MBL2* (ENST00000373968.3) from GRCh37/hg19, and in green the *LNCAROD_3* (ENST00000443523) from GENCODE V32.2 GRCh38/hg18. (*C*) Representation of the GeneHancer (GH) track from UCSC genome browser (enhancers ‐gray‐ and promoters ‐red‐). (*D*) SNPs associated with bone parameters in different genome‐wide association studies (GWASs), extracted from the GWAS catalog (https://www.ebi.ac.uk/gwas/).

We tested if the region containing 24 BMD‐GWAS SNPs, located 350‐kb downstream of *DKK1* (viewpoint, dashed gray line, in Fig. 3), is actually regulating *DKK1*, *MBL2*, or both. We performed a 4C‐seq experiment in three types of bone‐related cells (MSCs, hFOB 1.19, and the Saos‐2 osteosarcoma cell line), and we applied an algorithm to discern the significant contacts (see Materials and Methods; arched lines in Fig. 3). We only observed interactions of this viewpoint with sequences included in a genomic region spanning from 628 kb centromeric to the viewpoint to 141 kb telomeric. No other interactions were detected elsewhere in the genome. Inside this interval, we detected two main significant interacting regions common to all three cell types. The first one coincides with the *DKK1* promoter; it is the strongest signal detected. The second corresponds to an extended region including several *LNCAROD* transcripts and enhancer signals according to the GeneHancer database^(^ ^52^ ^)^(Fig. 3). No significant interaction was detected with *MBL2*, except for an interaction with sequences located several kb upstream of it in Saos‐2 cells. Altogether, we have concluded that the BMD‐GWA‐SNP‐rich region is interacting with both *DKK1* and the lncRNA *LNCAROD* in three bone‐related cell types.

## Discussion

In this work, we have studied the functionality of intragenic *DKK1* variants and analyzed a region harboring a cluster of genome‐wide significant SNPs from different GWASs. We have determined that four of six missense mutations affect the DKK1 inhibitory capacity. Additionally, we have determined that the intergenic region containing the genome‐wide significant signals interacts not only with *DKK1*, but also with the lncRNA *LNCAROD* gene in bone cells.

### Missense variants

**Fig 4:**
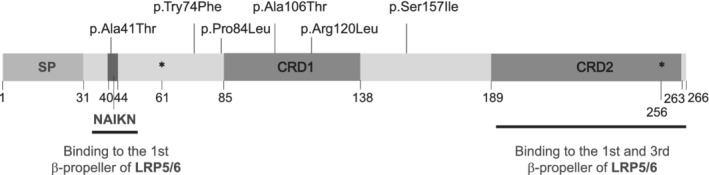
Domain structure of the DKK1 protein and position of the missense variants tested. The limits of the domains and the glycosylation sites (dots) are indicated below the scheme of the protein, together with the LRP5/6 binding domains (NAIKN motif and CRD2). CRD = Cysteine‐rich domain; SP = signal peptide.

We have tested the functionality of six DKK1 missense variants. Two of them were found in two women with HBM in previous studies of our group, p.Try74Phe^(^ ^53^ ^)^ and p.Arg120Leu^(^ ^35^ ^)^ and five, including the latter, are among the most frequent missense variants in the general population according to gnomAD v3 (p.Arg120Leu, p.Ala106Thr, p.Ser157Ile, p.Pro84Leu and p.Ala41Thr). To test their possible functionality, we have performed a luciferase reporter assay specific for the canonical Wnt pathway and we have determined that mutations p.Ala41Thr, p.Tyr74Phe, p.Arg120Leu and p.Ser157Ile affect DKK1 inhibitory capacity whereas p.Pro84Leu and p.Ala106Thr did not. Interestingly, none of the variants tested are located in the CRD2 domain (amino acids 189–263) known to bind to the LRP5 first and third β‐propeller to exert the inhibition (Fig. 4).

Variant p.Arg120Leu, which displays the strongest effect on DKK1 inhibitory capacity (55.2%), is the consistently predicted as deleterious, according the four most commonly used in silico tools (Table 1). In a previous work,^(^ ^35^ ^)^ we detected it in a woman with HBM (sum *Z*‐score = 6.20; menarche at 12 years; hysterectomy at 33 years; chronic mild renal insufficiency, arterial hypertension, and obesity). We propose that the loss‐of‐function of the protein with the p.Arg120Leu mutation may be contributing to the HBM phenotype of this woman, through insufficient inhibition of the Wnt pathway. In fact, this variant modifies the N‐terminal cysteine‐rich domain (CDR1; Fig. 4), described to exert independent signaling functions through other unknown receptors or pathways.^(^ ^55^ ^)^ The loss‐of‐function that we have observed for this variant does not agree with the study by Korvala and colleagues,^(^ ^56^ ^)^ who did not find a lower inhibitory capacity for the same mutated protein. Technical differences may explain these negative results. In fact, these authors and others^(^ ^56^, ^57^ ^)^ have found this mutation in patients with primary osteoporosis, a phenotype that does not fit with the loss of function of DKK1 and diametrically opposed to the HBM phenotype of our patient. Possible explanations for these discrepant findings may be a reduced penetrance for this variant and differences in patients' genetic background. Further investigation, preferably in vivo, may resolve this discrepancy.

**Table 1:** Pathogenicity Predictions of the Six Missense Variants Tested

Mutations	In silico predictors				MAF (gnomAD v3)	Loss of inhibitory capacity (%)	Sign.
	SIFT	PolyPhen‐2	CADD	REVEL			
p.Arg120Leu	D	PD	LD	LDC	0.00282	55.2	*
p.Ala41Thr	D	B	LB	LB	0.000128	37.8	*
p.Pro84Leu	T	PssD	LB	LB	0.000088	—	ns
p.Ser157Ile	T	B	LB	LB	0.000884	37.0	*
p.Try74Phe	T	B	LB	LB	0.0000201	34.0	*
p.Ala106Thr	T	B	LB	LB	0.00228	—	ns

Loss of inhibitory capacity and significance (Sign.) according to the results presented in this article. B = benign; CADD = combined annotation dependent depletion (available from: https://cadd.gs.washington.edu/; D = deleterious; gnomAD v3 = Genome Aggregation Database, version 3; LB = likely benign; LD = likely deleterious; LDC = likely disease causing; PD = probably damaging; MAF = minor allele frequency; ns = nonsignificant; PolyPhen‐2 = polymorphism phenotyping version 2 (available from: http://genetics.bwh.harvard.edu/pph2/); PssD = possibly damaging; REVEL = rare exome variant ensemble learner^(^ ^54^ ^)^; SIFT = sorting intolerant from tolerant (available from: https://sift.bii.a‐star.edu.sg/); T = tolerated.

* *p* < 0.001.

The p.Arg120Leu and also p.Ala41Thr have been described as associated with type I Chiari disease (CMI).^(^ ^58^ ^)^ This disease is defined as a downward herniation of the cerebellar tonsils across the foramen magnum caused by defects in the development of the occipital bone and the posterior fossa, which produces a neurologic dysfunction by direct compression of the neural tissue in the craniovertebral junction.^(^ ^59^, ^60^ ^)^ Given the bone involvement in CMI, it will be interesting to study the possible relationship between mutations in *DKK1* and this disorder, which in many cases is asymptomatic and undiagnosed.

p.Ala41Thr is the variant with the second highest loss of inhibitory capacity (37.8%) and also predicted as deleterious by SIFT software (Sorting Intolerant From Tolerant; available from: https://sift.bii.a-star.edu.sg/; Table 1). This variant affects the NAIKN motif (amino acids 40–44; Fig. 4), crucial for binding to the first β‐propeller of LRP5/6 proteins and present in all Wnt‐pathway inhibitors.^(^ ^20^ ^)^ Its frequency in the general population is 2.56 of every 10,000 individuals, but its effect in terms of bone mass is still unknown, as it happens for the p.Ser157Ile variant. The latter also displayed lower inhibitory activity, but in this case it may be a consequence of reduced secretion of the protein to the extracellular space (Supplementary Information Fig. S1). Finally, the variant p.Try74Phe was found in a previous study cosegregating in a family with HBM,^(53^ ^)^ and the functional effect identified here is consistent with the cosegregation in the HBM family.

### 3′UTR and splicing variants

In contrast to missense variants, we were not able to find functional evidence for variants rs74711339 and rs1569198, located in the 3′UTR and in intron 3, respectively. The effect of these variants in other cell types or in different developmental stages cannot be ruled out.

### GWAS hit locus

A group of 30 SNPs associated with bone parameters in 10 different GWASs exists in a 3.3‐Mb window in 10q21.1,^(^ ^1^, ^3^, ^4^, ^5^, ^36^, ^37^, ^38^, ^39^, ^40^, ^41^ ^)^ of which 24 are clustered within a 73‐kb genomic region located 320 kb telomeric from *DKK1* and 54 kb centromeric from *MBL2* (Fig. 3*D*). However, no enhancer marks overlap with this region, only two of these 24 SNPs are described as eQTLs (rs7088220 and rs12218358 for *MBL2*; GTEx v8) and no interactions between this GWA‐SNP–rich region and neighboring genes have been reported. We undertook a 4C‐seq assay to determine its interactions across the genome in three bone cell types, and found that the GWA‐SNP–rich region is interacting with both *DKK1* and regions that harbor several newly described transcripts of *LNCAROD*. Interestingly, this lncRNA has been recently shown to enhance *DKK1* transcription,^(^ ^61^ ^)^ requiring chromosomal proximity between the *A‐ROD* and the *DKK1* loci. Our 4C‐seq data are in agreement with these results because we found interactions with both *DKK1* and *LNCAROD*, which suggests a regulatory role for the GWA‐SNP–rich region. It should be noted that in the study by Ntini and colleagues,^(^ ^61^ ^)^ they only considered short forms of *LNCAROD*, whereas the last build of the genome (hg38) includes new longer versions, overlapping with the GWA‐SNP–rich region (see green lines in Fig. 3*B*). The regulatory role of the GWA‐SNP–rich region is further supported by the doubleridge spontaneous mouse. This mouse model bears a 60‐kb deletion (located 150 kb from *Dkk1* and 90 kb from *Mbl2*), probably corresponding to this human region, and displays a drastic drop in *Dkk1* expression levels (between 35% and <1% of WT), while keeping the levels of neighboring *Mbl2* and *Prkg1* genes intact.^(^ ^28^, ^29^ ^)^ In this sense, it would be interesting to verify if *LNCAROD* is underexpressed in this mouse model, which would confirm the role of this lncRNA in *DKK1* regulation. Our results clearly show for the first time that this region is relevant for the regulation of *DKK1*, and it will be very interesting to assess the specific role of the different SNPs in this process.

### Limitations

This work has two main limitations. One of them is the complex luciferase assay used for the study of missense mutations. This method involves the cotransfection of several vectors, which implies high variability. It is possible that some of the variants tested, expected to have a small effect, show differences in activity below the sensitivity of the reporter gene assay we have used. Regarding the eQTL study, the main limitation is the sample size. Because of difficulties in obtaining appropriate samples, we only were able to use 45 primary osteoblasts, which precluded testing SNPs with low allele frequency. It would be useful to have a bank of primary osteoblasts with a much higher sample size to get information on different eQTLs important for bone; this information is lacking in current databases.

## Conclusions

In conclusion, for the first time we show functional evidence for the relevance of *DKK1* in HBM phenotype and osteoporosis determination. Four missense variants in DKK1 affect its inhibitory capacity, consistent with the HBM phenotype of a few individuals bearing these changes. Additionally, genomic interactions exist between a region rich in BMD‐GWA‐SNP and the *DKK1* gene, and also with a lncRNA known to enhance *DKK1* expression. These functional data open a way to study the mechanisms of *DKK1* regulation, which may define novel therapeutic targets for bone diseases.

## Disclosures

Service on Advisory Board: DO: Kyowa Kirin; XN: Amgen, UCB; ADP: Amgen, UCB.

Honoraria or royalties for books or publications or for lectures (speaker fees), or participating in a speakers bureau: DO: Speaker fees in a couple of talks for Kyowa Kirin; XN: Speaker fees from Amgen, Lilly; ADP: Lilly, Amgen, UCB, Gilead, Theramex.

Research grants, direct salary support or other financial support from commercial entities: DO: Research grant funded by Kyowa Kirin.

Stock holdings and/or stock options in pharmaceutical, medical: ADP: Active Life Sci.

The other authors declare no conflicts of interest.

## Author Contributions

**Núria Martínez‐Gil:** Conceptualization; data curation; formal analysis; investigation; methodology; writing‐original draft; writing‐review and editing. **Neus Roca‐Ayats:** Investigation; methodology; writing‐review and editing. **Nurgül Atalay:** Investigation; methodology; writing‐review and editing. **Marta Pineda‐Moncusí:** Data curation; formal analysis; writing‐review and editing. **Natalia Garcia‐Giralt:** Investigation; methodology; writing‐review and editing. **Wim van hul:** Conceptualization; writing‐review and editing. **Eveline Boudin:** Investigation; methodology; writing‐review and editing. **Diana Ovejero:** Investigation; methodology; writing‐review and editing. **Leonardo Mellibovsky:** Investigation; methodology; writing‐review and editing. **Xavier Nogués:** Investigation; methodology; writing‐review and editing. **Adolfo Diez‐Perez:** Investigation; methodology; writing‐review and editing. **Daniel Grinberg:** Conceptualization; funding acquisition; supervision; writing‐original draft; writing‐review and editing. **Susanna Balcells:** Conceptualization; funding acquisition; supervision; writing‐original draft; writing‐review and editing.

## Data accessibility statement

The software package and the 4C data files used to make the statistical analysis, are publicly available at https://github.com/Nikoula86/AFourC.git. All data and material will be available upon request.

### Peer Review

The peer review history for this article is available at https://publons.com/publon/10.1002/jbm4.10423.

## Supporting information


**Appendix** S1: Supplementary Information
