# Supplementary material for: Functional Assessment of Coding and Regulatory Variants From the DKK1 Locus
Source: JBMR Plus. 2020 Nov 2;4(12):e10423. doi: 10.1002/jbm4.10423 (PMC7745885; doi:10.1002/jbm4.10423)
Supplement: Supplementary file 1 — Appendix S1: Supplementary Information [file JBM4-4-e10423-s001.docx]

**Functional assessment of coding and regulatory variants from the *DKK1* locus**

Núria Martínez-Gil^1^, Neus Roca-Ayats^1^, Nurgül Atalay^1^, Marta Pineda-Moncusí^2^, Natàlia Garcia-Giralt^2^, Wim Van Hul^3^, Eveline Boudin^3^, Diana Ovejero^2^, Leonardo Mellibovsky^2^, Xavier Nogués^2^, Adolfo Díez-Pérez^2^, Daniel Grinberg^1^, Susanna Balcells^1^

1 Department of Genetics, Microbiology and Statistics, Faculty of Biology, Universitat de Barcelona, CIBERER, IBUB, IRSJD, Barcelona, Spain.

2 Musculoskeletal Research Group, IMIM (Hospital del Mar Medical Research Institute), Centro de Investigación Biomédica en Red en Fragilidad y Envejecimiento Saludable (CIBERFES), ISCIII, Barcelona, Spain.

3 Center of Medical Genetics, University of Antwerp & University Hospital Antwerp, Antwerp, Belgium.

**Running Title:** *DKK1* coding and regulatory variants

**Corresponding author:**

Susanna Balcells: [sbalcells@ub.edu](mailto:sbalcells@ub.edu).

Dpt. Genètica, Microbiologia i Estadística

Facultat Biologia

Av. Diagonal, 643

08028 Barcelona

Tel +34 93 4035418

| **Table 1. Sequence of all the primers used** | |  |
| --- | --- | --- |
| **Experiment** | **Primer name** | **Sequence (5'-->3')** |
| pmirGLO-rs74711399 | rs74711339_F | ctcgagAAACCAGCTATCCAAATGCAG |
|  | rs74711339_R | cctgcaggCAATCACAGGGGAGTTCCAT |
| seq-eQTLs | rs1569198 and rs74711339 F | AGGTGCTGCACTGCCTATTT |
|  | rs1569198 and rs74711339 R | CCGTATCCTCATTCCAATCAA |
|  | rs1373004_F | GAGCCACTTGATTAATGTCTCTGA |
|  | rs1373004_R | TTGATTAAACAAAAGGTTCACAAA |
| Site directed mutagenesis | p.Ala41Thr_F | GGGCAGGTTCTTGATAGTGTTGGAATTGAGAACCG |
|  | p.Ala41Thr_R | CGGTTCTCAATTCCAACACTATCAAGAACCTGCCC |
|  | p.Try74Phe_F | CCCGGGCGGGAATAAGTTCCAGACCATTGACAAC |
|  | P.Try74Phe_R | GTTGTCAATGGTCTGGAACTTATTCCCGCCCGGG |
|  | p.Pro84Leu_F | GTCCTCTGCGCACAGGTACGGCTGGTA |
|  | p.Pro84Leu_R | TACCAGCCGTACCTGTGCGCAGAGGAC |
|  | p.Ala106Thr_F | TTTGCACGCCTGTGTCCCCTCCGCG |
|  | p.Ala106Thr_R | CGCGGAGGGGACACAGGCGTGCAAA |
|  | p.Arg120Leu_F | CGTGACGCATGCAGAGTTTTCGGCGCTTC |
|  | p.Arg120Leu_R | GAAGCGCCGAAAACTCTGCATGCGTCACG |
|  | p.Ser157Ile_F | GTGCTATGATCATTACCAAAGATTTCAGTGATGGTTTCCTCAA |
|  | p.Ser157Ile_R | TTGAGGAAACCATCACTGAAATCTTTGGTAATGATCATAGCAC |
| DKK1_qPCR | DKK1_qPCR_F | CTCCCGGACCCTGACTCT |
|  | DKK1_qPCR_R | ACAAAGACCCGGGTAGCTC |
| HBMS_qPCR | HMBS_qPCR_F | TGCCCTGGAGAAGAATGAAG |
|  | HMBS_qPCR_R | CAGCATCATGAGGGTTTTCC |

| **Table 2.** cis-eQTL information from GTEX database for the rs7471133 and rs1569198 SNPs. | | | |
| --- | --- | --- | --- |
| **SNP** | **Gene** | **P value** | **Tissue** |
| **rs74711339** | *DKK1* | 2.2e-10 | [Cells cultured fibroblasts](about:blank) |
| **rs1569198** | *PRKG1-AS1* | 2.5e-10 | [Cells cultured fibroblasts](about:blank) |
|  | *DKK1* | 2.8e-5 | Adrenal-Gland |


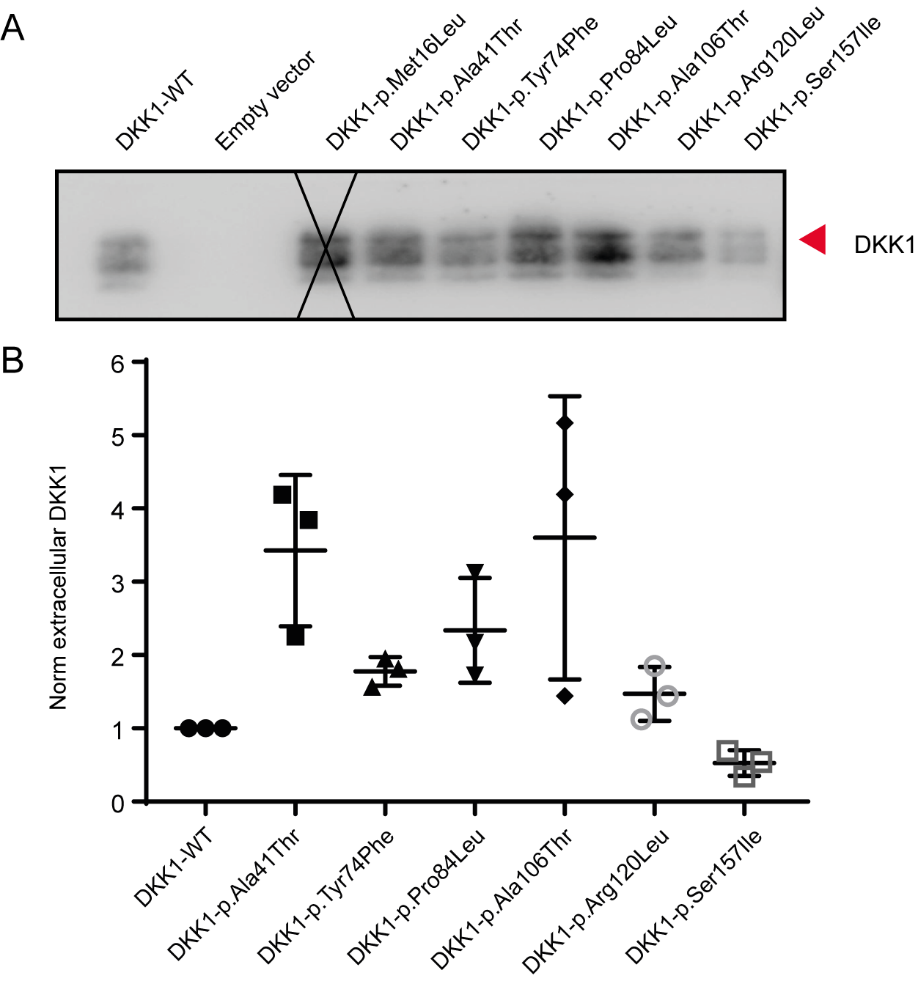


**Fig.1. Extracellular expression of six DKK1 missense variants.** A) Western blot of heterologously expressed DKK1 proteins secreted to the extracellular space. Crossed-out lane: mutation unrelated to this work. B) Quantification of three western blot replicates normalized to the expression levels of DKK1-WT.


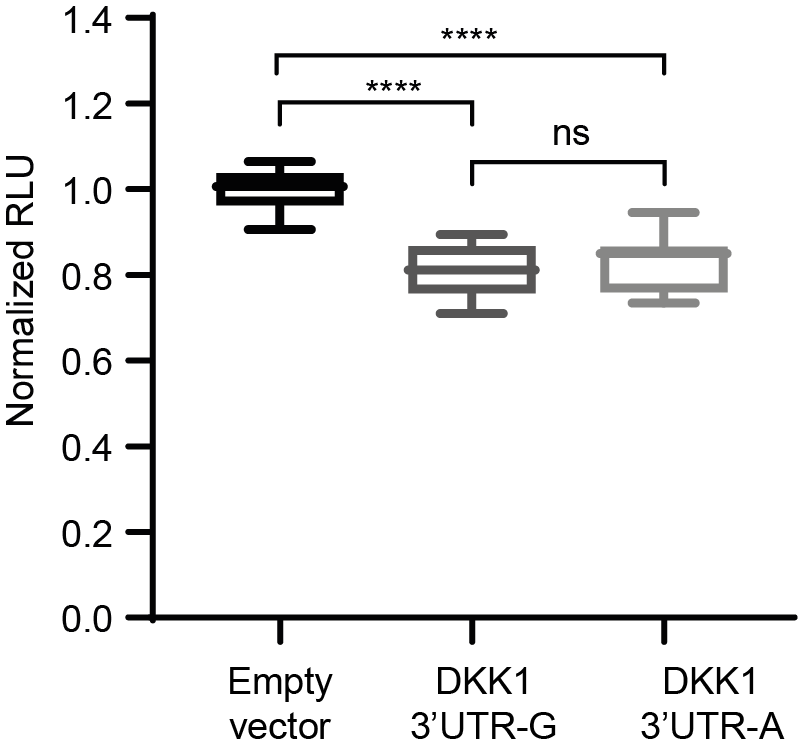


**Fig. 2.** **Luciferase assays of the DKK1 3’UTR region.** Boxplots of the normalized relative luciferase activity of the pmirGlo vector (*Empty vector*) and the constructs with the 3’UTR region of *DKK1*-containing the rs74711339 G and A alleles. Significant differences indicated as ****p<0.0001. Error bars represent the SD.


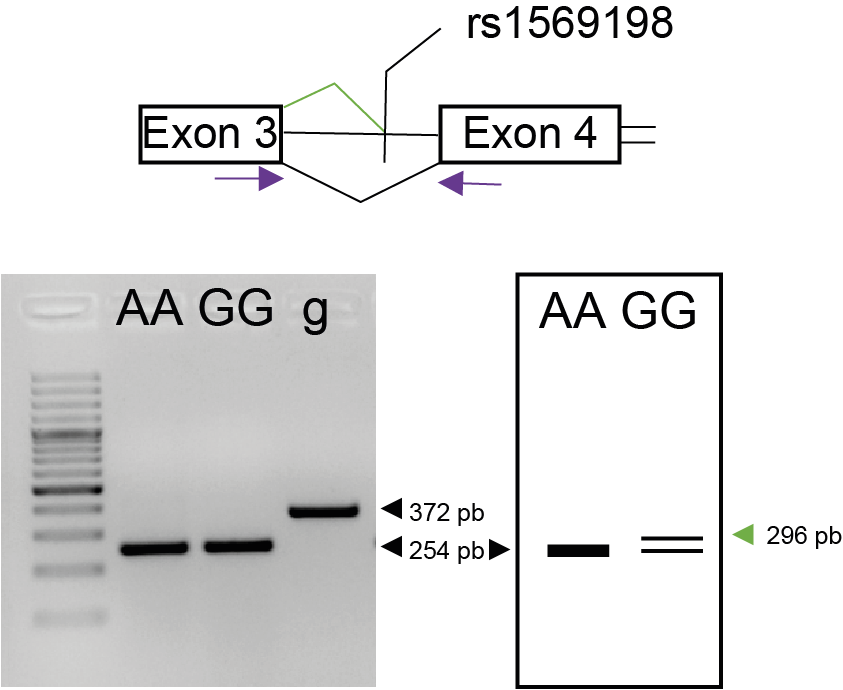


**Fig. 3.** RT**-**PCR amplification for hOB from homozygous women of each of the alleles of the SNP rs1569198. Top: Scheme intron 3 and flanking exons indicating the primers binding site (arrows) and the SNP position. Left: Representative electrophoresis of the RT-PCR products for the genotypes AA and GG all of 254 bp. Right: Result prediction under the hypothesis of a partial use of the alternative splice site (296 pb) generated by the G allele. g: PCR amplification of the genomic DNA to display an amplicon of 372 pb which includes the complete intron 3.


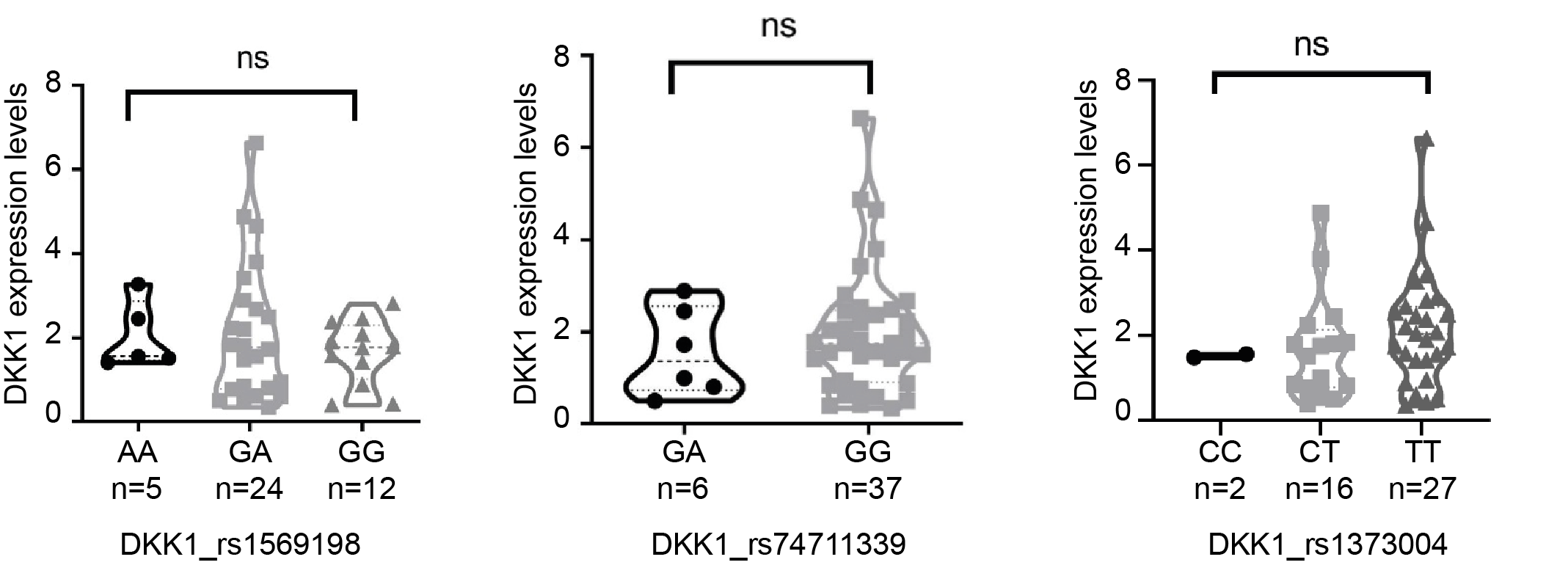


**Fig. 4.** Violinplot of *cis*-eQTL analysis of SNPs rs1569198, rs74711339 and rs1373004 on *DKK1* expression levels in hOB (n=45). ns: no significant
